# Learning curve for lateral lymph node dissection in rectal cancer – a systematic review of literature

**DOI:** 10.1007/s10151-025-03214-3

**Published:** 2025-10-25

**Authors:** D. Kehagias, L. Baldari, E. Cassinotti, L. Boni, C. Lampropoulos, I. Kehagias

**Affiliations:** 1 Department of Surgery, University of Patras, Patras, Greece; 2 Department of General and Minimally Invasive Surgery, Fondazione IRCCS Ca’ Granda Ospedale Maggiore Policlinico, Milan, Italy; 3 Department of Scienze Cliniche e delle Comunità, University of Milan, Milan, Italy; 4 Department of Fisiopatologia Medico-Chirurgica e dei Trapianti, University of Milan, Milan, Italy; 5 Intensive Care Unit, Saint Andrew’s General Hospital of Patras, Patras, Greece

**Keywords:** Lateral lymph node dissection, Learning curve, Rectal cancer, Lateral lymph nodes

## Abstract

**Background:**

Lateral lymph node dissection (LLND) remains controversial owing to differences in oncological principles between East and West, complex pelvic anatomy and the risk of complications. The aim of this systematic review is to determine the number of cases required to achieve surgical competence in LLND and to evaluate postoperative outcomes across different phases of the learning curve.

**Methods:**

A systematic literature search was conducted in PubMed and Google Scholar for studies analyzing the LLND learning curve in rectal cancer resection. The three-phase pattern, consisting of learning, competence, and proficiency, was followed for data analysis and presentation. A separate learning curve analysis for open, laparoscopic and robotic LLND was performed. Blood loss, operative time, lymph node yield, urinary complications and postoperative morbidity were assessed across the phases of the learning curve for robotic LLND.

**Results:**

Of the 616 articles screened, eight studies met the inclusion criteria. Seven studies reported the learning curve analysis for robotic LLND, and one study for laparoscopic and open approach. Five studies had operative time as a learning outcome, two studies the lymph node yield and one study both lymph node yield and urinary retention. All studies used the cumulative sum (CUSUM) method for learning curve analysis. Regardless of learning outcome, surgical competence for robotic LLND was achieved after 12–53 cases, for laparoscopic LLND after 19 cases, and for the open approach no inflection point was identified. In robotic LLND, blood loss, urinary complications, and morbidity decreased during the proficiency phase.

**Conclusions:**

The LLND learning curve is not yet standardized owing to variability in study design, type of LLND, and learning outcomes. Further well-designed and methodologically consistent studies are required to establish learning benchmarks and improve patient outcomes.

**Registration in PROSPERO database:**

CRD420251050015.

## Introduction

Lateral lymph node dissection (LLND) in rectal cancer remains a subject of intense controversy, reflecting important differences in oncological principles between the East and the West [1]. In Western countries, lateral lymph nodes (LLN) are considered as a systemic disease, and neoadjuvant chemoradiotherapy (nCRT) with total mesorectal excision (TME) is the mainstay of treatment. Conversely, in the East, particularly in Japan, these nodes are regarded as locoregional disease, requiring surgical removal [2]. These differing perspectives have impeded the global standardization and widespread implementation of LLND in rectal cancer management.

An increasing body of high-quality evidence supports the selective incorporation of LLND, particularly in patients with radiologically suspicious or positive LLNs. The landmark trial JCOG012 demonstrated that in stage II–III rectal cancer patients, who did not receive nCRT and had LLNs with a short-axis diameter of < 10 mm, LLND with TME significantly decreased local recurrence compared with TME alone [3]. A subsequent subgroup analysis further revealed that patients with stage III disease, who underwent TME with LLND exhibited a significantly increased 7-year recurrence-free survival, compared with TME alone [4]. Additionally, data from the Lateral Node Study Consortium, involving 1216 patients, showcased the importance of lymph node size. LLNs measuring ≥ 7 mm were associated with a lateral local recurrence rate of 19.5%, despite treatment with nCRT and TME. In contrast, patients who underwent LLND following nCRT exhibited a substantially lower recurrence rate of 5.7% in similarly sized nodes [5]. Notably, the same study found that patients with LLNs ≥ 7 mm, who responded well to nCRT, achieving a size of below 4 mm, could potentially avoid LLND, without compromising oncological outcomes [6]. These findings suggest that nCRT alone may not be sufficient for eradicating microscopic metastases in the LLNs, thereby reinforcing the selective use of LLND.

Despite this evidence, a lot of skepticism and reluctance to perform LLND persists among surgeons. Many clinicians advocate that LLND without nCRT might constitute overtreatment, while others question the validity of existing data revolving around LLND in the context of total neoadjuvant therapy (TNT) [7]. Another significant contributor to this hesitation is the limited familiarity with the complex operative field of the lateral pelvis. The anatomical complexity of this region makes the procedure technically challenging, while it is potentially associated with increased intraoperative blood loss, prolonged operative time, urogenital dysfunction, postoperative morbidity, and incomplete lymph node clearance [8]. To overcome these challenges, minimally invasive techniques have been implemented. Robotic platforms offer enhanced dexterity in the deep narrow pelvis, while advanced surgical navigation tools, such as indocyanine green, stereotactic or three-dimensional (3D) holographic navigation, are increasingly being used, improving precision [9, 10]. Minimally invasive approaches accompanied by a comprehensive understanding of the pelvic anatomy, particularly the areas of the obturator and internal iliac nodes, are mandatory for ensuring a safe and complete LLND [11]. Nevertheless, LLND remains a demanding procedure with a steep learning curve, requiring effort and dedication to achieve surgical competence [12].

Learning curves depict the relationship between learning effort and the selected learning outcome, represented on the x and y axes, respectively. Originally developed in industrial settings, learning curves were used to demonstrate how the number of man-hours required for the manufacture of an aircraft decreased with increasing production volume [13]. In surgery, they were implemented in the 1980s to describe the acquisition of skills required for minimally invasive procedures [14]. It is generally accepted that the learning curve follows a characteristic pattern: an initial phase of rapid learning and improvement, followed by a plateau phase indicating competence, and ultimately a phase of mastery or proficiency [15]. The phase of decline of competence, usually owing to overconfidence or “forgetting”, is also described in some models. Several confounding factors can affect the shape and interpretation of a learning curve, including prior experience, individual background, and the availability of appropriate equipment. Moreover, many studies adopt a descriptive analysis of the learning curve, limiting the precision of the conclusions. A variety of methods have been employed to assess a learning curve, ranging from graphical inspection and split-group comparisons, to cumulative sum (CUSUM) and regression analysis [16]. Despite certain limitations, learning curves are a useful tool for evaluating surgical competence, guiding the development of training curricula, and ensuring the safe and effective implementation of new procedures. The aim of this systematic review is to assess the number of cases required to reach competence for LLND in rectal cancer resection and to compare postoperative outcomes across different phases of the learning curve.

## Methods

### Search strategy

A systematic literature review was conducted in accordance with the Preferred Reporting Items for Systematic Reviews and Meta-Analyses (PRISMA) guidelines [17]. The electronic databases Google Scholar® and PubMed® (National Library of Medicine, Bethesda, MD, USA) were searched for articles published until May 2025. The systematic review was registered in the PROSPERO database (UIN: CRD420251050015).

The search strategy included a combination of keywords and Medical Subject Headings (MeSH) terms, with Boolean operators. The following search syntax was utilized: “lateral pelvic lymph node dissection” OR “lateral lymph node dissection” OR “lateral lymph node” AND “learning curve” AND “rectal”. The advanced search syntaxis can be found in the protocol registered in the PROSPERO database. Two authors independently performed the literature search across both databases. Duplicate articles were removed prior to screening. Following this, irrelevant studies were excluded by reviewing titles and abstracts. Additionally, the references of the retrieved articles were also examined to identify additional studies, not captured in the initial search. A third author served as a supervisor and resolved any disagreements regarding study selection.

The retrieved articles were rigorously evaluated for eligibility. Studies were included if they reported learning curve analysis of LLND for rectal cancer. Inclusion criteria were as follows: (1) articles published in English, (2) prospective or retrospective studies assessing the learning curve for LLND, and (3) studies clearly describing LLND as the dissection of the internal iliac and obturator nodes. The exclusion criteria were: (1) editorials, commentaries, or conference abstracts, (2) non-English language publications, (3) books or book chapters, (4) systematic or narrative reviews, (5) studies not addressing the learning curve for LLND (6) studies focusing on learning curves for procedures other than LLND in rectal cancer surgery, and (7) case studies or case series.

### Data extraction and definitions

For each included study, two authors extracted the following data: author, year of publication, country, and study design. Information regarding the number of patients and the type of rectal resection performed in conjunction with LLND was also collected.

Additionally, variables that could potentially affect the learning curve were extracted. These included the number of surgeons contributing to the learning curve and their previous surgical experience. The type of LLND in each study, bilateral or unilateral, was also recorded, since it may influence the interpretation of the learning curve. Ultimately, the surgical approach used, either open, laparoscopic or robotic, was also documented.

Regarding learning curve analysis, several parameters were extracted. These included the method used to assess the learning curve, the distinct phases of the learning curve, the learning outcome and the number of required cases. Where reported, outcomes and complications of LLND for each phase were extracted and included in the analysis. Specifically, data regarding operative time, blood loss, lymph node yield, urinary complications and postoperative morbidity were collected. Urinary complications were used as a wider definition, including urinary retention, prolonged postoperative catheter use, and the need for a-agonist medications. Postoperative morbidity was defined as any complication on the basis of Clavien–Dindo classification or as any major complication, defined as Clavien–Dindo ≥ III.

### Data synthesis and analysis

A standardized three-phase learning curve, consisting of learning, competence, and proficiency, is supported by several studies in minimally invasive surgery [18–20]. Accordingly, this pattern was applied in studies that described three phases, ensuring consistency of data. In studies reporting only two phases, these were classified as the learning and competence phases. In studies outlining four phases, there were two learning phases, followed by competence and proficiency phases.

Studies that assessed learning curves in mixed cohorts, where not all patients underwent LLND, were analyzed separately. When a sub-analysis of patients with LLND was available, these data were extracted and reported independently. Furthermore, studies evaluating the learning curve for open or laparoscopic LLND were presented separately from those focusing on robotic LLND, to reflect differences in surgical approach.

Studies providing details of the outcomes and complications, and making comparisons between the phases of the learning curve, were separately analyzed. The data and the comparisons were extracted from each study. Values were expressed as mean ± standard deviation (SD) or median ± interquartile range (IQR), while *p* value was < 0.05 for significance. Comparisons between phases of the learning curve were recorded as presented in each individual study.

### Quality assessment

Two authors independently assessed the risk of bias of the included studies, using the new version of the risk of bias in non-randomized studies—of interventions, Version 2 (ROBINS – I V2) tool, which was available in November 2024 [21]. This tool evaluates seven domains and uses structured algorithms to generate risk of bias assessment for each domain, as well as an overall rating. A third author was involved in discussing and resolving any discrepancies.

## Results

### Screening process and characteristics of included studies

**Fig. 1:**
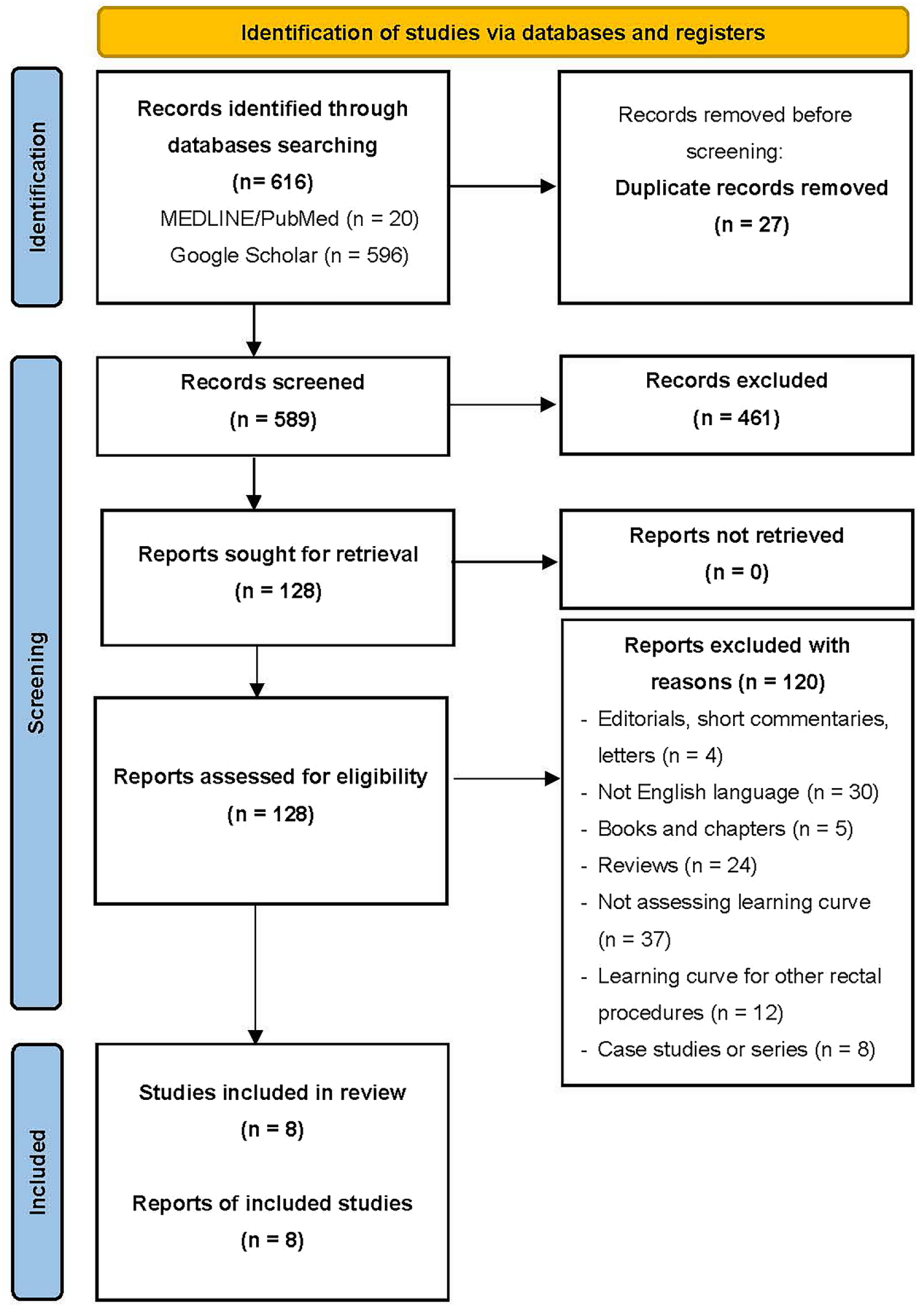
Prisma Flowchart

**Fig. 2:**
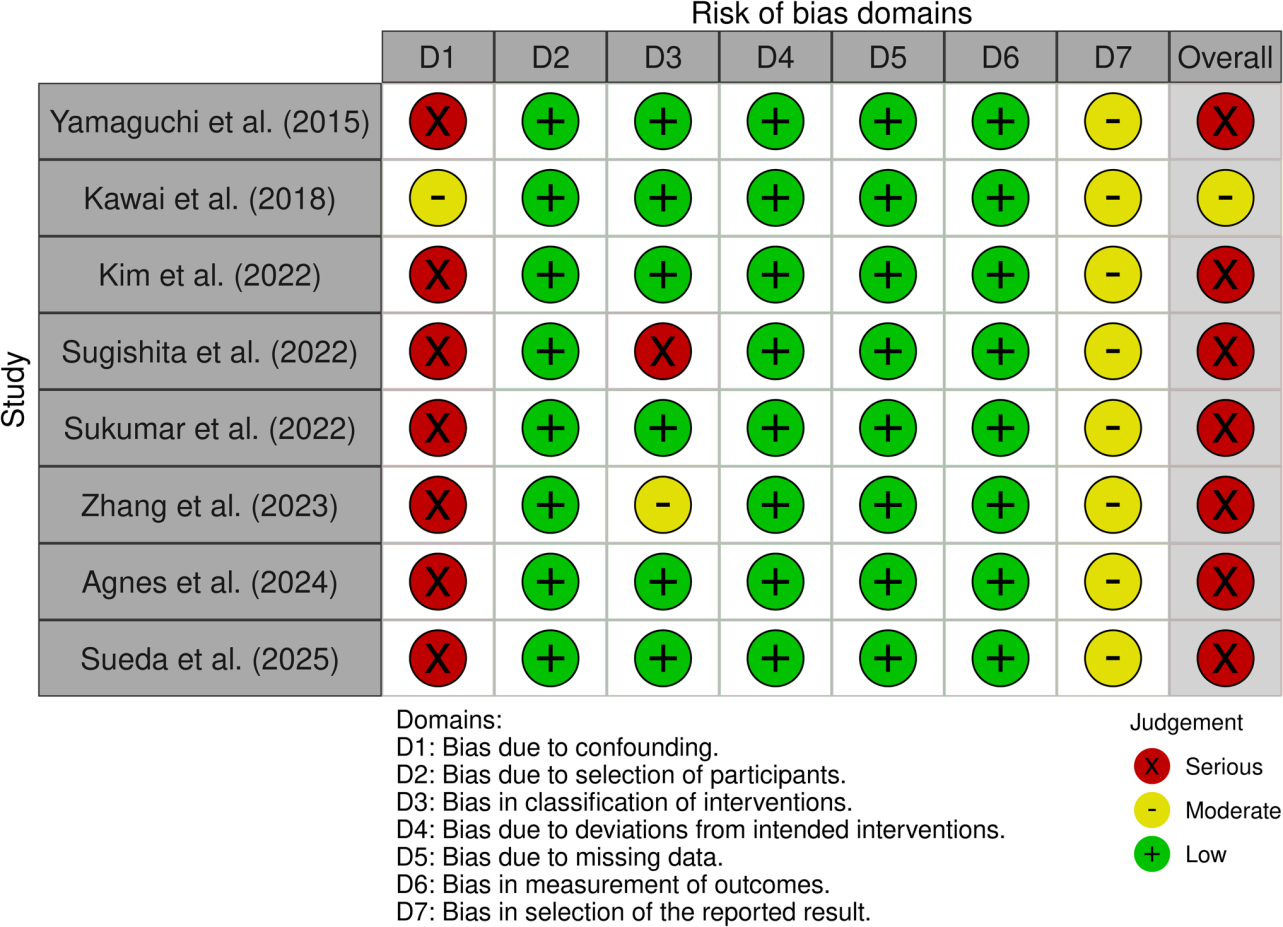
Risk of bias assessment with ROBINS – I V2 tool

After the review process, eight studies were deemed eligible and were included in the analysis [22–29]. The PRISMA flowchart (Fig. 1) depicts the systematic process used to identify and select studies for inclusion. According to the ROBINS-I V2 tool, seven studies had a serious risk of bias, and one had moderate risk. Overall, the risk of bias across the included studies was judged to be serious, as illustrated in Fig. 2.

All the included studies analyzing the learning curve for LLND were retrospectively designed and 87.5% (7 out of 8) were conducted in Eastern institutions. Among the Eastern studies, over half (4 out of 7) were conducted in Japan, with one study each from China, India, and South Korea. In contrast, only one study originated from a Western institution, specifically in the USA [28].

In terms of surgical approach, LLND was performed robotically in 87.5% of the studies (7 out of 8), while one study analyzed learning curves for both laparoscopic and open approaches. In nearly all studies, LLND was part of a rectal resection, involving mostly low anterior resection, followed by inter-sphincteric resection and abdominoperineal resection. Only one study included five cases where LLND was performed as a stand-alone procedure [28].

A total of 504 patients were included in the learning curve analysis. In five studies, all patients underwent LLND, whereas three studies included mixed cohorts, presenting patients who underwent rectal resections with or without LLND [22, 23, 25].

In 62.5% of the studies (5 out of 8), the LLND procedures of the learning curve were performed by a single surgeon. The remaining studies involved multiple surgeons (two, three and four, respectively). In the first four chronologically studies, published from 2015 to 2022, surgeons had expertise in open and laparoscopic surgery, but limited robotic experience. Conversely, the four most recent studies, published from 2022 to 2025, featured surgeons with established robotic experience and previous exposure to LLND. All included studies (100%) employed the CUSUM method to assess the learning curve (Table 1).

**Table 1:** Characteristics of included studies

Author	Year	Country	Study design	Included procedures (*n*)	Patients with LLND *n* (%)	Surgeons *n*	Previous experience of surgeons	Learning curve analysis
Yamaguchi et al. [22]^2^	2015	Japan	Retrospective	R—AR (6) R—LAR (46) R—ISR (22) R—APR (6)	28/80 (35%)	1	Expert in laparoscopic > 500 RC resections No robotic experience	CUSUM
Kawai et al. [23]	2018	Japan	Retrospective	R—AR (6) R—LAR (94) R—ISR (30)	41/130 (31.5%)	1	Expert in laparoscopic > 500 RC resections No robotic experience	CUSUM
Kim et al. [24]	2022	Korea	Retrospective	R—LAR (50) R—ISR (45) R—APR (5)	100/100 (100%)	1	Expert in open/laparoscopic No robotic experience	CUSUM
Sugishita et al. [25]	2022	Japan	Retrospective	R—AR (9) R—LAR (103) R—ISR (28) R—APR (9)	25/149 (16.7%)	1	90 cases of laparoscopic RC resections No robotic experience	CUSUM
Sukumar et al. [26]	2022	India	Retrospective	Open/Lap—LAR (44) Open/Lap—APR (58) Open/Lap—PE (18)	120/120 (100%)	2	Experts in laparoscopic/robotic 10 – 15 LLND for RC > 50 LLND for other pelvic	CUSUM
Zhang et al. [27]	2023	China	Retrospective	R—LAR (40) R—ISR (17) R—APR (21)	78/78 (100%)	1	400 laparoscopic and 100 robotic cases per year Limited experience in LLND	CUSUM
Agnes et al. [28]	2024	USA	Retrospective	R—LAR (33) R—APR (16) R—LLND only (5)	54/54 (100%)	4	Two experts in robotic RC resections Two proctored for LLND	CUSUM
Sueda et al. [29]	2025	Japan	Retrospective	R—LAR (39) R—ISR (1) R—APR (18)	58/58 (100%)	3	≥ 5 open/laparoscopic LLND ≥ 40 robotic RC resections	CUSUM

*R–AR* robotic anterior resection, *R–LAR* robotic low-anterior resection, *R–ISR* robotic inter-sphincteric resection, *R–APR* robotic abdominal-perineal resection, *LLND* lateral lymph node dissection, *RC* rectal cancer, *CUSUM* cumulative sum;

### Learning curve analysis in rectal resections with and without LLND

Three studies included mixed patient cohorts in which not all individuals underwent robotic LLND [22, 23, 25]. Of these, two studies performed sub-analyses focusing specifically on the learning curve for patients who received LLND [23, 25]. In all three studies, the learning outcome for the learning curve was the operative time, while only in one study the type of LLND was clarified, including bilateral and unilateral cases [25].

In the study that did not perform sub-analysis, 25 cases were required to reach competence, from which only 13 involved LLND. After 50 cases, proficiency was achieved; hence, only 23 cases included LLND.

Two studies focused only on the cohort of patients with LLND combined with TME, presenting a sub-analysis of the learning curve. Kawai et al. presented two phases in the learning curve, with competence achieved after 21 cases [23]. Sugishita et al. included both bilateral and unilateral cases of LLND and reported that competence was reached after 12 cases (Table 2).

**Table 2:** Learning curve analysis in studies including rectal resections with and without LLND

Author	Learning outcome	Surgical approach	Types of LLND	LLND learning curve assessment	Phases of learning curve, range of cases			Learning curve outcome, n cases
Yamaguchi et al. [22]	Operative time	Robotic	.	Not separate analysis of LLND cases	Learning 1–25 13/25 LLND*	Competence 26–50 10/25 LLND*		Proficiency 51–80 5/30 LLND*
Kawai et al. [23]	Operative time	Robotic	.	Sub-analysis LLND + TME	Learning 1–21	Competence 22–41		21
Sugishita et al. [25]	Operative time	Robotic	Bilateral Unilateral	Sub-analysis LLND + TME	Learning 1–12	Competence 13–18	Proficiency 19–25	12

*LLND* lateral lymph node dissection, *TME* total mesorectal excision

*Only the indicated number of patients in each phase underwent LLND

### Learning curve analysis for open and laparoscopic LLND

One study investigated the learning curve for open and laparoscopic approaches to LLND [26]. A total of 120 patients were included, with 64 undergoing laparoscopic LLND and 56 undergoing open LLND. Both bilateral and unilateral LLND were included, and all these cases were performed between 2014 and 2021. The learning outcome assessed in both approaches was the lymph node yield. An inflection point in the learning curve was identified only in the laparoscopic cohort. Specifically, the authors described two phases and 19 cases were required to reach competence. Conversely, no inflection point or distinct phases of the learning curve were identified in the open surgery cohort (Table 3).

**Table 3:** Learning curve analysis for laparoscopic and open LLND

Author	Surgical approach	Number of patients	Period performed	Types of LLND	Learning outcome	Phases of learning curve, range of cases		Learning curve outcome, n cases
Sukumar et al. [26]	Laparoscopic	64	2014–2021	Unilateral Bilateral	LLND yield	Learning 1–19	Competence 20–64	19
	Open	56	2014–2021	Unilateral Bilateral	LLND yield			None inflection point

*LLND* lateral lymph node dissection

### Learning curve analysis in studies including only patients with robotic LLND

Four studies analyzed the learning curve in patient cohorts that underwent robotic LLND [24, 27–29]. One of these studies reported two separate learning curves, one single-surgeon and one institutional learning curve [28]. Three studies included both unilateral and bilateral LLND cases, while one study exclusively analyzed bilateral prophylactic LLND cases [29].

The learning outcomes varied across studies. Two studies used operative time, one used the lymph node yield, and one study presented dual learning curves, employing lymph node yield and postoperative urinary retention as learning outcomes [28].

Regarding the trend of the learning curves, two studies described three distinct phases, while one identified four phases, including two learning phases [24, 27, 29]. In the study that presented both institutional and single-surgeon learning curves, two phases were primarily observed, except in the analysis of the institutional learning curve focused on lymph node yield, which demonstrated a three-phase pattern [28].

When lymph node yield was used as the primary learning outcome, the number of cases required to achieve competence ranged from 12 to 53. For studies using operative time, achieving competence required 20–28 cases. When urinary retention was assessed as the learning outcome, competence was reached after 20–26 cases (Table 4).

**Table 4:** Learning curve analysis in robotic LLND

Author	N of patients with LLND	Period performed	Types of LLND	Learning outcome	Phases of learning curve, range of cases				Learning curve outcome, *n* cases
Kim et al. [24]	100	2011–2017	Unilateral Bilateral	LLND yield	Learning I 1–33	Learning II 34–52	Competence 53–82	Proficiency 83–100	53
Zhang et al. [27]	78	2015–2021	Unilateral Bilateral	Operative time	Learning 1–28	Competence 29–46	Proficiency 47–78		28
Agnes et al. [28]	35 (single—surgeon)	2012–2015	Unilateral Bilateral	LLND yield	Learning 1–12	Competence 12–35			LLND yield 12–13 urinary retention 20–26
				Urinary retention	Learning 1–20	Competence 21–35			
	54 (institutional)	2012–2021	Unilateral Bilateral	LLND yield	Learning 1–13	Competence 14–26	Proficiency 27–54		
				Urinary retention	Learning 1–26	Competence 27–54			
Sueda et al. [29]	58	2020–2024	Bilateral	Operative time	Learning 1–20	Competence 21–36	Proficiency 37–58		20

*LLND* lateral lymph node dissection

### Outcomes across learning curve phases in studies of robotic LLND

Across the four studies focusing exclusively on patients undergoing robotic LLND, progressive improvements in surgical outcomes were observed during the learning curve phases [24, 27–29]. These included reductions in blood loss, urinary complications, and overall postoperative morbidity (Table 5).

**Table 5:** Outcomes between the learning phases in studies including only robotic LLND cases

Author	Outcomes	Phases of learning curve (n of cases)				*P* value
		Learning I (*n* = 33)	Learning II (*n* = 19)	Competence (*n* = 30)	Proficiency (*n* = 18)	
Kim et al. [24]	Operative time, unilateral LLND (min)	46.0 ± 9.7 ^†^	49.8 ± 7.4 ^†^	53.5 ± 5.7 ^†^	49.9 ± 3.5 ^†^	> 0.05
	Blood loss, unilateral LLND (ml)	37.5 ± 29.3 ^†^	43.5 ± 23.1 ^†^	47.2 ± 29.3 ^†^	27.5 ± 39.6 ^†^	** < 0.05 **^**a**^
	LLND yield, unilateral	4.9 ± 3.4 ^†^	8.2 ± 4.1 ^†^	10.4 ± 4.9 ^†^	12.8 ± 4.7 ^†^	** < 0.05 **^**b, c**^
	Urinary complications, *n* (%)	13 (39.4)	4 (21.1)	6 (20.0)	3 (16.7)	** < 0.05 **^**c**^
	Postoperative morbidity within 90-d, n (%)	11 (33.3)	5 (26.3)	10 (33.3)	4 (22.2)	> 0.05

Author	Outcomes	Learning (*n* = 28)	Competence (*n* = 18)	Proficiency (*n* = 32)		*P* value
Zhang et al. [27]	Operative time, unilateral LLND (min)	38 (27–49) ^‡^	34 (23–44) ^‡^	29 (19–41) ^‡^		** < 0.05 **^**d**^
	Blood loss, unilateral LLND (ml)	25 (5–100) ^‡^	20 (5–40) ^‡^	15 (5–45) ^‡^		** < 0.05 **^**d**^
	LLND yield, unilateral	6 (2–15) ^‡^	6 (2–12) ^‡^	7 (1–14) ^‡^		> 0.05
	Urinary complications, *n* (%)	8 (28.6)	4 (22.2)	6 (18.8)		> 0.05
	Postoperative morbidity, *n* (%)	9 (32.1)	5 (27.8)	6 (18.8)		> 0.05

Author	Outcomes	Learning (*n* = 13)	Competence (*n* = 13)	Proficiency (*n* = 28)		*P* value
Agnes et al. [28]	Operative time (min)	.	.	.		
	Blood loss (ml)	.	.	.		
	LLND yield, unilateral	4 ± 3 ^†^	7 ± 5 ^†^	6 ± 4 ^†^		
	Urinary complications, n (%)	4 (30.8%)	4 (30.8%)	3 (10.7%)		
	Major complications, n (%)	2 (15.4%)	2 (15.4%)	2 (14.3%)		

Author	Outcomes	Learning (*n* = 20)	Competence (*n* = 16)	Proficiency (*n* = 22)		*P* value
Sueda et al. [29]	Operative time, bilateral LLND (min)	187 ± 28.8 ^†^	173.3 ± 27.9 ^†^	160.5 ± 14.9 ^†^		** < 0.05 **^**e**^
	Blood loss, bilateral LLND (ml)	183 ± 168.0 ^†^	105 ± 181.2 ^†^	40.5 ± 47.5 ^†^		** < 0.05 **^**e**^
	LLND yield, bilateral	37 ± 13.2 ^†^	29 ± 5.8 ^†^	30.5 ± 7.6 ^†^		> 0.05
	Urinary complications, *n* (%)	5 (25.0)	0 (0.0)	0.0 (0.0)		** < 0.05 **^**e**^
	Clavien–Dindo classification, all grade, *n* (%)	8 (40)	6 (37.5)	1 (4.5)		** < 0.05 **^**e**^

^**†**^Values expressed as mean ± standard deviation (SD); ^‡^Values expressed as median and interquartile range (IQR); ^a^Competence vs proficiency; ^b^Learning I vs II; ^c^Learning I vs competence; ^d^Learning vs proficiency; ^e^Learning and competence vs proficiency; *p* < 0.05 and bold for significance

In three studies, intraoperative blood loss was significantly decreased during the proficiency phase compared with the earlier phases [24, 27, 29]. The remaining study did not report blood loss data [28]. Urinary complications were significantly lower in the proficiency phase in two studies (*p* < 0.05) [24, 29]. In the other two studies, a downward trend in urinary complication rates was observed across the learning curve phases, though without reaching statistical significance [27, 28]. Postoperative morbidity significantly decreased in one study during the proficiency phase [29]. In the other three studies, morbidity showed a declining trend across the learning curve phases, but the changes were not statistically significant [24, 27, 28].

Operative time was significantly reduced in the studies where it was used as learning outcome [27, 29]. In contrast, studies that used outcomes of lymph node yield or urinary retention, reported no significant change in operative time across the phases [24, 28].

Regarding the lymph node yield, in two studies it remained unchanged among the different phases of the learning curve [27, 29]. However, in one study that had as a learning outcome the lymph node yield, a significant increase was observed from the learning to the competence phase [24]. In the remaining study, also using lymph node yield as a primary outcome, higher yields were reported during the competence and proficiency phases compared with the learning phase [28].

## Discussion

LLND is still not an established procedure worldwide, largely owing to differing oncological practices between Eastern and Western countries. This controversial nature has contributed to limited surgeon familiarity and a scarcity of robust data in literature. This systematic review found that the learning curve for LLND is not yet well-defined, with no clear benchmarks or guidance on the duration of proctoring. Most available studies focus on robotic LLND, with reported case numbers required to achieve competence ranging from 12 to 53, while only one study addressed the learning curve for open and laparoscopic approach. Considerable variability exists in reported learning outcomes, with operative time being the most commonly assessed learning outcome, followed by lymph node yield and urinary retention. Several confounding factors were noted, including the number of surgeons involved, their prior experience, and the inclusion of both unilateral and bilateral LLND cases, resulting in a serious overall risk of bias. Nevertheless, reduced complication rates were observed as surgeons progressed from learning to competence, and particularly proficiency, during robotic LLND.

Regardless of the learning outcome, the number of cases required to achieve surgical competence in LLND varies by approach. For robotic LLND, 12 to 53 cases are needed, depending on the outcome measured, whereas laparoscopic LLND requires approximately 19 cases. No inflection point was identified for open LLND. Specifically, in robotic LLND, for lymph node yield, operative time and urinary retention, surgical competence was achieved after 12–53 cases, 12–28 cases, and 20–26 cases, respectively. Excluding the study by Kim et al. the range for robotic LLND narrows significantly to 12–28 cases, regardless of the learning outcome. Kim et al. described two learning phases, encompassing a total of 52 cases. Notably, during the second learning phase, there was an increase in lymph node yield and a reduction in urinary complications, potentially indicating achievement of surgical competence [24]. In laparoscopic LLND, Sukumar et al. observed an increase in lymph node yield after the 19th case, suggesting competence. In contrast, no clear learning curve was identified for open LLND. As the authors explained, there are limitations during open LLND, since the internal iliac region is very low in the narrow pelvic space [26]. This further emphasizes the importance of minimally invasive surgery when working in the deep narrow pelvis and particularly the robotic approach, which offers enhanced dexterity and precision [30].

Numerous variables are used as learning outcomes among studies investigating the learning curve. These are categorized into (1) measures of the surgical process, such as operative time, conversion rate, blood loss, outcomes of resection margins and lymph node yield; (2) measures of patient outcome including postoperative complications, morbidity, functional outcomes and oncological outcomes [31]. Operative time is the most commonly used learning outcome among studies in minimally invasive surgery [16]. This was verified in this systematic review, where five studies used it to assess the learning curve. Although it is easy to obtain, its clinical relevance is debatable, since the reduction of operative time at the expense of patient outcomes should not be considered a marker of success [32]. Additionally, in some studies analyzing the learning curve, operative time fails to decline with experience and this often reflects surgeons’ increased confidence and willingness to undertake more complex cases after the initial learning phase [33]. For instance, in the included study by Kim et al. operative time failed to decrease after the initial learning phase. This could be explained by the higher rate of neoadjuvant treatment in patients after the initial learning phase, as well as the use of other modalities such as indocyanine fluorescence imaging for guidance, which increased the duration of the procedure [24]. Furthermore, the definition of operative time often varies, particularly in robotic surgery, where distinctions are made between total operative time and surgeon console time [34].

Following operative time, lymph node yield was the second most commonly assessed learning outcome. The lymph node yield indicates the quality of the performed LLND, which is considered a standardized technique. The dissection of the internal iliac and obturator compartments involves three anatomical planes (1) medial plane: ureter and pelvic plexus—ureterohypogastric fascia (2) lateral plane: adjacent to the psoas and internal obturator internus muscle and (3) dorsal plane: internal iliac vessels and the sciatic nerve [12]. Given the standardized nature of the procedure, one would expect lymph node yield to remain relatively consistent across different phases of the learning curve. Indeed, only one study by Kim et al. reported a significantly increased number of harvested lymph nodes after the initial learning phase. However, the cases by Kim et al. were performed between 2011 and 2017, while the surgeon had no previous experience with robotic surgery and LLND, compared with the more contemporary studies [24]. Additionally, adequate LLND needs to be translated into terms of oncological outcomes, since a higher number of retrieved lymph nodes does not necessarily mean more positive nodes. In the same study by Kim et al., the number of positive lateral lymph nodes was equal across all phases. Additionally, Sueda et al. suggested that retrieved LLNs should always be categorized into internal iliac and obturator regions during learning curve analysis, since the obturator region has a higher lymph node yield [29]. However, further evidence needs to be furnished to understand the role of lymph node yield and lymph node regions during LLND and how these affect the oncological outcomes.

As with operative time, the use of lymph node yield as a learning outcome is subject to several confounding factors. Most studies in the Eastern institutions report the number of unilateral lymph nodes retrieved during LLND [28]. However, this was not clear in this systematic review as most studies included cases with unilateral and bilateral LLND. This significantly complicates the interpretation of the learning curve, as performing bilateral LLND in a single patient effectively reduces the number of individual cases needed to reach surgical competence. Only one study by Sueda et al. exclusively assessed prophylactic bilateral LLND cases. This methodological consistency strengthens their findings, suggesting that competence is achieved after 20 cases [29]. Moreover, several other variables may influence lymph node yield. The use of nCRT can reduce nodal size, potentially affecting yield. In addition, the pathologist’s experience and learning curve may also impact the accuracy of lymph node retrieval and reporting [28, 35]. Finally, patient-related factors, such as body mass index (BMI), can influence the technical difficulty of dissection within the deep, narrow pelvis, further affecting nodal yield [27].

Therefore, utilizing patient outcomes as learning outcomes could be more meaningful than operative time and lymph node yield for evaluating the learning curve. Nevertheless, patient outcomes are dichotomous variables, such as postoperative complications or survival, which might occur infrequently and thus require large sample sizes for analysis [31]. Despite this limitation, patient outcomes are considered more reliable as learning outcomes for assessing surgical performance [28, 36]. Only one study by Agnes et al. utilized urinary retention, demonstrating the need of 20 to 26 cases to achieve surgical competence [28]. However, it was not possible to determine whether the incidence of urinary retention was influenced by the type of surgical procedure associated with LLND, such as low anterior resection or abdominoperineal resection.

As most studies assessed the learning curve during robotic LLND, the outcomes during the different phases were evaluated. Overall, a decrease in complications rates, including blood loss, urinary complications and morbidity, was observed, particularly during the proficiency phase. While some patient characteristics such as ASA score and tumor stage were comparable between learning curve phases, other factors, such as BMI and nCRT, were not consistently balanced across studies, introducing a potential selection bias [24, 27, 28]. Furthermore, the definition of postoperative morbidity varied, including major complications, 90-day morbidity or the use of Clavien–Dindo classification. Although most studies focus on Clavien–Dindo grade III and above, only one included study by Sueda et al. stratified complications by grade. Their analysis demonstrated a significant reduction in overall complication rates across learning phases, but no significant difference in Clavien–Dindo grade III complications [29, 37].

An important consideration when interpreting learning curve data is the impact of the surgical setting, prior experience, and the number of surgeons involved in each study. Institutions with pre-existing expertise in beyond-TME resections and robotic surgery are likely to demonstrate shorter and more stable learning curves, benefiting from the crossover effect and structured proctoring, which promote early standardization and consistency in outcomes [38]. This was evident in the study by Agnes et al., where a relatively stable performance was observed when new surgeons started performing the procedure within a controlled, proctor-supported environment [28]. Additionally, a surgeon’s prior operative experience has consistently been shown to influence the learning curve. Soomro et al. reported that greater cumulative surgical experience correlates with a faster acquisition of competence in robotic procedures [32]. Shu et al. found that surgeons with extensive laparoscopic experience who underwent a structured robotic training curriculum achieved shorter operative times, likely owing to improved anatomical orientation and decision-making [39]. All of the above support the broader concept of institutional learning, in which not only the individual surgeon, but the entire surgical team evolves. These findings underscore the importance of viewing the learning curve as a multidisciplinary phenomenon, encompassing the collective adaptation of both surgeon and team [38].

This systematic review has several important limitations. Although it was conducted in accordance with PRISMA guidelines and utilized PubMed and Google Scholar to capture both peer-reviewed and grey literature on LLND learning curves, it did not incorporate other major bibliographic databases, such as Embase and the Cochrane Library. Consequently, relevant studies may have been missed, potentially introducing selection bias. Furthermore, all included studies were single-center and retrospective in design, which inherently carries a high risk of selection bias and may have influenced both the reported outcomes and the interpretation of the learning curve. Learning outcomes demonstrated substantial variability across studies, indicating a lack of uniform criteria for learning curve assessment. This heterogeneity reflects the absence of standardized metrics for evaluating surgical learning in LLND. Unlike the JCOG0212 trial—which employed photographic verification to standardize the extent of lateral dissection—none of the included studies described the use of intraoperative or postoperative audit protocols to verify the completeness of the dissection. Additionally, considerable variation existed in the number of surgeons involved, their prior experience with minimally invasive surgery and LLND, the degree of proctoring, and the overall institutional proficiency. These factors likely contributed to inconsistencies in the reported learning curves. Several potential confounding variables—including BMI, nCRT, laterality of LLND (unilateral versus bilateral), and the indication for LLND (therapeutic versus prophylactic)—were inadequately reported or not addressed in most studies, further complicating the interpretation of learning outcomes. Although a limited meta-analysis of operative time and lymph node yield for robotic LLND was considered, the four available studies reported outcomes using disparate endpoints and summary statistics (e.g., means ± standard deviation versus medians and interquartile ranges; total operative time versus LLND-specific time). This level of methodological heterogeneity rendered quantitative synthesis statistically inappropriate. Overall, these limitations introduce a considerable risk of bias, as assessed using the ROBINS-I Version 2 tool, and underscore the methodological inconsistencies across the existing literature. Nevertheless, this review represents the first comprehensive synthesis of current evidence on the learning curve associated with LLND, contributing valuable insight into a technically demanding and debated surgical procedure.

There is a clear need for high-quality, methodologically robust studies to better elucidate the learning curve associated with LLND during rectal cancer surgery. An important phenomenon is the tendency to perform fewer complex cases early in the learning phase, followed by more challenging cases in the intermediate phase. This progression results in case-mix heterogeneity, which can distort the accuracy of learning curve assessments [40]. To address this, the use of risk-adjusted cumulative sum (RA-CUSUM) analysis is suggested. This advanced statistical method adjusts for confounding variables, that may influence operative performance, thus providing a more accurate representation of a surgeon’s progression over time. However, the effective application of RA-CUSUM requires large datasets, which remains a challenge given that LLND is not yet widely adopted globally [41, 42]. For future research, we recommend the incorporation of quality control measures—such as standardized photographic or video-based assessments employing predefined anatomic landmarks—to ensure consistent and objective validation of surgical technique. Clinically meaningful endpoints should extend beyond technical surrogates and include long-term oncologic outcomes, such as lateral pelvic recurrence-free survival and overall survival, as well as patient-reported outcomes related to urinary and sexual function, standardized complication grading, and overall health-related quality of life. Moreover, future studies should directly compare perioperative outcomes during and after the learning curve to determine whether the early learning phase is associated with increased morbidity. Such investigations should preferably be conducted in high-volume centers with established expertise in minimally invasive colorectal surgery and beyond-TME procedures to enhance both generalizability and methodological robustness. Finally, to facilitate future meta-analyses, we recommend that subsequent studies on the LLND learning curve adopt standardized definitions of outcome measures, report key data consistently (including means, variances, or confidence intervals), and use harmonized phase classifications, thereby enabling rigorous subgroup analyses and reliable data synthesis.

## Conclusions

LLND is still not an established procedure and the data regarding the learning curve is currently limited. The majority of existing studies focus on robotic LLND, reporting a range of 12 to 53 cases required to achieve surgical competence. However, differences in patient-related and surgeon-related factors are drawbacks, contributing to variability in the analysis of the learning curve. This systematic review highlights the need for well-designed, prospective, multicenter studies using standardized outcome measures, to better define the learning curve of LLND and the development of consensus on benchmarking parameters. This could be beneficial in enhancing surgical training programs worldwide, enabling surgeons to achieve proficiency more efficiently and safely, without compromising patient outcomes.

## Data Availability

No datasets were generated or analyzed during the current study.
